# Asymmetric light reflectance from metal nanoparticle arrays on dielectric surfaces

**DOI:** 10.1038/srep18331

**Published:** 2015-12-18

**Authors:** K. Huang, W. Pan, J. F. Zhu, J. C. Li, N. Gao, C. Liu, L. Ji, E. T. Yu, J.Y. Kang

**Affiliations:** 1 Fujian Provincial Key Laboratory of Semiconductors and Applications, Collaborative Innovation Center for Optoelectronic Semiconductors and Efficient Devices, Department of Physics, Xiamen University, Xiamen 361005, P. R. China; 2 Department of Chemistry, State Key Laboratory of Physical Chemistry of Solid Surfaces, Xiamen University, Xiamen 361005, China; 3 Department of Electrical and Computer Engineering, Microelectronic Research Centre, The University of Texas at Austin, Austin, Texas, 78758, USA

## Abstract

Asymmetric light reflectance associated with localized surface plasmons excited in metal nanoparticles on a quartz substrate is observed and analyzed. This phenomenon is explained by the superposition of two waves, the wave reflected by the air/quartz interface and that reflected by the metal nanoparticles, and the resulting interference effects. Far field behavior investigation suggests that zero reflection can be achieved by optimizing the density of metal nanoparticles. Near field behavior investigation suggests that the coupling efficiency of localized surface plasmon can be additionally enhanced by separating the metal NPs from substrates using a thin film with refractive index smaller than the substrate. The latter behavior is confirmed via surface-enhanced Raman spectroscopy studies using metal nanoparticles on Si/SiO2 substrates.

Localized surface plasmons (LSPs), the charge density oscillations confined in metallic nanostructures, have attracted tremendous interest for a broad range of emerging applications, such as chemical and biomolecular sensing^1^,^2^,^3^,^4^, subwavelength optical imaging^5^,^6^, optoelectronic devices such as light emitting diodes (LEDs)^7^,^8^,^9^, solar cells^10^,^11^,^12^,^13^,^14^ and photodetectors^15^,^16^,^17^,^18^. LSPs excited by an electric field at a particular incident wavelength where resonance occurs will lead to strong light scattering, intense absorption band and enhancement of the local electromagnetic fields^19^, The oscillation frequency and intensity i. e. coupling efficiency of LSPs can be modulated by the type of metals^20^. They are also highly sensitive to the size, size distribution, shape and the medium which surround/near the metallic nanostructure^21^,^22^.

In this work, we characterize and analyse asymmetric light reflectance observed for metal nanoparticles (NPs) fabricated on quartz substrate. Far field reflection spectra show different behaviours at wavelengths close to the LSP resonance wavelength when light is normally incident from air compared to that when light is incident through the quartz substrate. This phenomenon can be explained using a modified Fresnel coefficients model^23^,^24^,^25^. Specifically, the far field reflected wave can be regarded as a superposition of the wave reflected by the air/quartz interface and that reflected by the LSPs. The reflection-phase shift of LSPs is π at the LSP resonance wavelength, so the superposition leads to either constructive or destructive interference when light is incident on the air/NPs/quartz interface from air or quartz, respectively. Theoretical analysis combined with FDTD simulations show that the reflection intensity at the LSP resonance wavelength can be reduced close to zero by varying the density of Au NPs. This behaviour can be used to enhance the sensitivity of LSP based sensors.

Theoretical analysis and FDTD simulation also indicate that when light is incident from quartz, the extinction peak intensity of the LSPs at the wavelength close to LSP resonance wavelength is larger than that when light is incident from air. This phenomenon can be attributed to the different local driving field intensities of LSPs when light is incident from different media. The ratio of the extinction peak intensities when light is incident from different media is equal to the ratio of the refractive indices of the two media. However, for many LSPs applications, light must be incident from air. Theoretical analysis and Surface-enhanced Raman scattering (SERS) measurements demonstrate that when metal NPs are separated from a substrate by a thin film with refractive index lower than the substrate, the local driving field intensity can be adjusted. The local driving field intensity can therefore be optimized by including a thin film with optimized thickness. This behaviour provides a general method to enhance the LSPs coupling efficiency that may improve the performance of the LSPs based devices for a variety of applications.

## Results

### Far field behaviour

**Figure 1:**
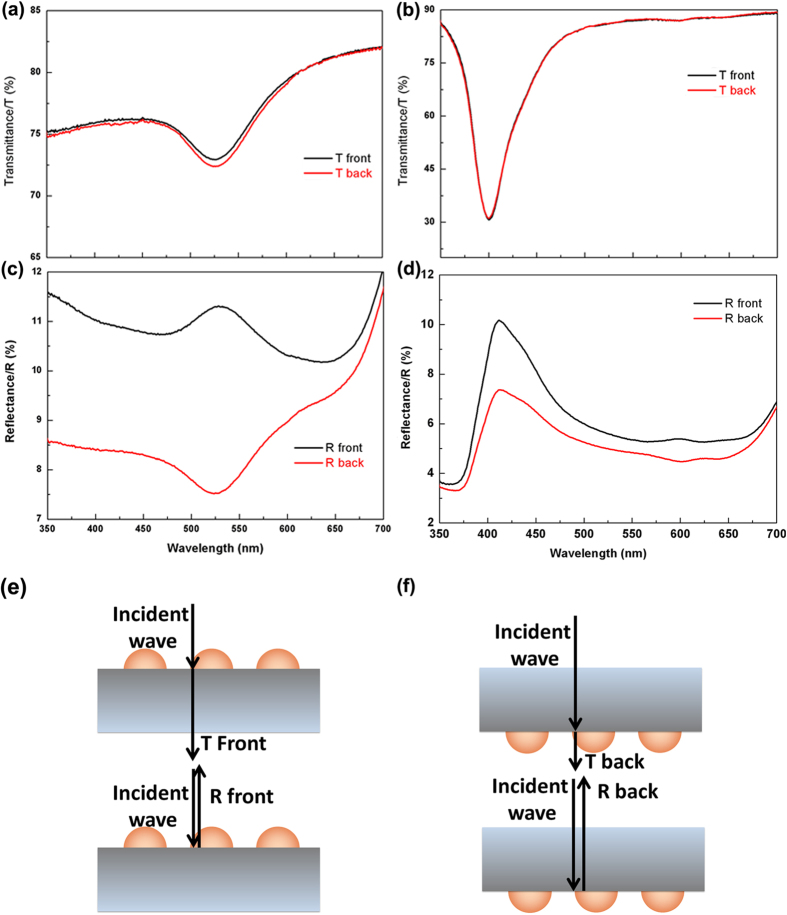
Far field behavior of metal NPs on quartz. (**a**,**b**) Transmittance spectra of Au and Ag NPs on quartz substrates. (**c,d**) Reflectance spectra of Au and Ag NPs on quartz substrates. (**e,f**) Schematic illustrations of transmittance and reflectance measurements when light incidence is from front and back sides.

Figure 1(a–d) present the far field transmittance and reflectance spectra, respectively, of Au and Ag NP arrays on a quartz substrate. One can see from Fig. 1(a) that when light is incident normally from air to the air/Au NPs/quartz interface (designated as front incident), the transmission spectrum shows a valley at approximately 525 nm corresponding to the LSP resonance mode of the Au NPs. When light is incident normally from the quartz substrate (designated as back incident), the transmission spectrum is almost the same as that of front incident, agreeing with the principle of reciprocity. Similarly, a dip in transmittance near the LSP resonance wavelength and nearly identical transmission spectra for front and back incidence are observed for Ag nanoparticles, as seen in Fig. 1(b). However, the reflectance spectra when light is incident from different directions differ substantially, as shown in Fig. 1c for the structure with Au nanoparticles on quartz. At the LSP resonance wavelength, the reflectance spectrum measured from the front side shows a peak while the spectrum measured from the back side shows a valley. For the sample consisting of Ag NPs on quartz substrate, peaks are seen in the reflectance spectra when light is incident from both sides. However, when light is incident from back side, the peak intensity is smaller than that when light is incident from front side (Fig. 1d).

The behavior seen in Fig. 1 can be explained using a model based on modified Fresnel coefficients^23^. This model take into account excess current and charge densities present due to the discrete subwavelength metal NPs at the interface. For a plane wave propagating through a medium with refractive index *n*_*i*_ toward an interface consisting of metal NPs against a medium with refractive index *n*_*t*_, the reflection coefficient *r* for a normally incident wave can be written as


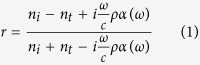


Here, 
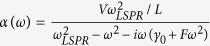
, where *V* is the volume of the NPs, 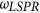 is the LSP resonance frequency, and *L* is a geometrical depolarization factor calculated by the image dipole theory^23^,^26^. For a hemisphere structure, one can obtain 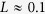. 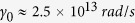 are the width of resonance determined by the resistive Drude damping factor^25^ and the factor 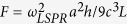 describes the radiative damping contribution that arises due to the finite size of the particle. Thus, one can see that when the frequency of the incident wave is equal to the LSP resonance frequency, *i.e.* 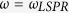, the additional term arising from the localized surface plasmon, 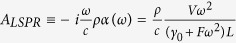, is a positive real number. Equation (1) can then be written as


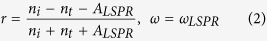


One can see that when 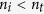 which corresponds to the front incident situation, we obtain 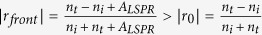, where 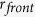 is the reflection coefficient when light is normally incident from the front side, 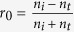 is the reflection coefficient for normally incident light when there is not any subwavelength metal NPs located at the interface. Thus reflectance 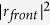 is greater than 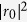, which means that the reflectance spectrum measured from the front side exhibits a peak at wavelengths close to the localized surface plasmon resonance wavelength.

When 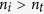, which corresponds to the back incident situation, we obtain 
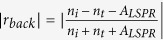
, where 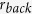 is the reflection coefficient when light is normally incident from the back side. One can see that the reflectance spectrum does not always show valley when light is incident from the medium with high refractive index. When 
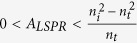
, we see that 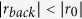, and the reflectance spectrum will exhibit a valley at wavelengths close to the localized surface plasmon resonance wavelength as shown in Fig. 1(c). When 
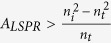
, we see that 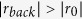, and the reflectance spectrum will contain a peak as shown in Fig. 1(d). However, regardless of the value of *A*_*LSPR*_, 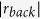 is smaller than 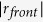. Thus when light is incident from the back side, the reflectance at wavelengths close to the localized surface plasmon resonance wavelength is smaller than that when light is incident from the front side.

**Figure 2:**
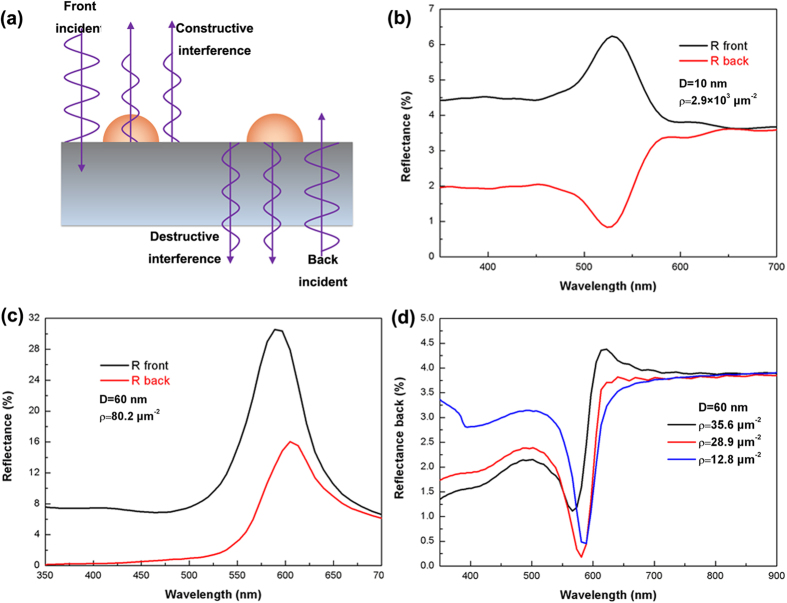
Mechanism and far field FDTD simulation. A schematic illustration of the asymmetric light reflectance phenomenon (**a**) and the simulated reflectance spectra for an air/quartz interface with hemispherical Au NPs located at the interface with different diameters and densities (**b,c**). The diameters and densities of Au NPs are 10 nm, 2.9 × 10^3^ μm^−2^ and 60 nm, 80.2 μm^−2^ respectively. (**d**) The simulated reflectance spectra of Au NPs on quartz at various densities when light is incident from back side, the densities of Au NPs are 35.6, 28.9 and 12.8 μm^−2^ respectively.

Figure 2 illustrates the mechanism leading to the asymmetric light reflectance phenomenon described above. For a plane wave propagating through a medium with refractive index *n*_*i*_ toward an interface consisting of metal NPs against a medium with refractive index *n*_*t*_, the reflected wave can be regarded as superposition of two waves: the reflected wave from the interface without metal NPs on it and the reflected wave arising from the metal NPs. Thus the reflection coefficient arising from the metal NPs can be written as


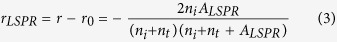


From Equation (3) one can see that 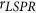 is a negative real number which indicates that a-phase shift of π is introduced when light is reflected by metal NPs. When light is incident from the medium with lower refractive index, the superposition of the two reflected waves leads to constructive interference. Thus the reflectance spectrum always shows a peak at wavelengths close to the localized surface plasmon resonance wavelength. When light is incident from the medium with higher refractive index, however, the superposition of the two reflected waves leads to destructive interference. Thus, when *A*_*LSPR*_ is not too large, the reflectance spectrum exhibits a valley rather than a peak at wavelengths close to the localized surface plasmon resonance wavelength (Fig. 2a). One can see that, to a certain degree, this phenomenon is similar to the asymmetric light reflectance effect we have reported previously in AAO on glass^27^. However, the phenomenon observed here still has some major differences from previous report, which are described as follows. (i) This phenomenon is wavelength selective and can be regulated by the resonance wavelength of the LSPs. (ii) The two superposing waves come from metal NPs and dielectric interface which attach to each other rather than two separated interfaces. (iii) Metal nanostructure is not necessarily to be embedded into an optical film. Thus it is more suitable for further near field applications.

One can see both the volume and density of metal NPs will affect the value of *A*_*LSPR*_ and therefore the reflectance intensity. If the diameter of the metal NPs is relatively small, peaks will not be present in the reflectance spectra when light is incident from back side even with relatively high NPs density. Figure 2b shows the simulated reflectance spectra of Au NPs (10 nm in diameter) with density as high as 2.9 × 10^3^ μm^−2^, the reflectance spectra show peak and valley when light is incident from front and back sides respectively. When the diameter of metal NPs is relatively large, the far field behavior can be tuned by varying the densities of the NPs. In particular, when 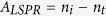, the reflection coefficient is zero at the the LSP resonance wavelength. Since *A*_*LSPR*_ is a positive real number, zero reflectance can happen only when light is incident from the medium with high refractive index. Figure 2c–d show the simulated reflection spectra with different Au NPs densities. One can see that when the density of Au NPs (60 nm in diameter) equals to 80.2 μm^−2^; the reflection spectra show peaks regardless the incident directions (Fig. 2c). For the large Au NPs with relatively low densities, the reflectance spectra show valleys when light is incident from the back side (Fig. 2d). One can see that when the density of Au NPs increases, the valley intensity decreases close to zero first and then increases. When the density of Au NPs equals to 28.9 μm^−2^, the reflection intensity at the LSP resonance wavelength can be as low as 0.15%.

### Near field behavior

**Figure 3:**
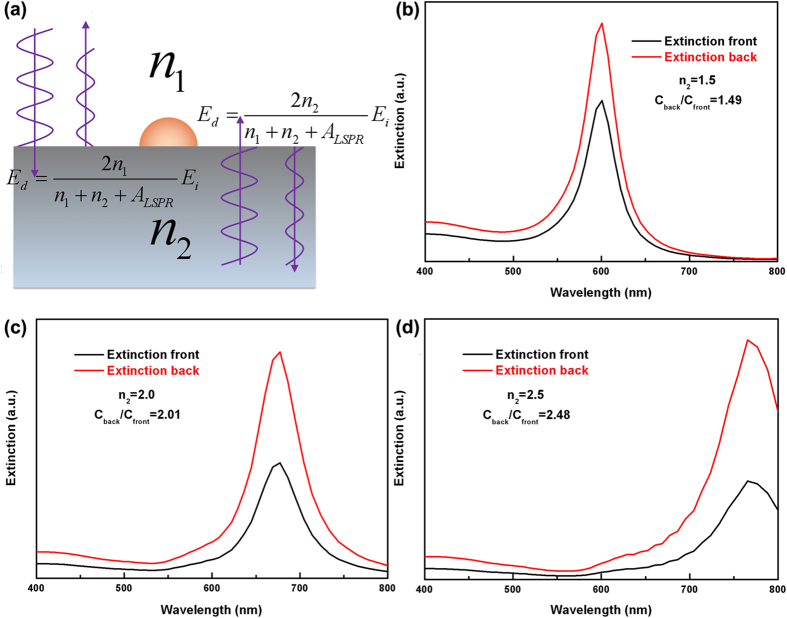
Local driving field intensities of LSPs and near field FDTD simulations. (**a**) A schematic illustration of local driving field intensities of LSPs. (**b–d**) Extinction spectra of Au NPs on substrates with various refractive indices when light is incident from air/substrate.

Equation (3) also indicates that when light is incident from media with different refractive indices, the reflection coefficients associated with the metal NPs are not the same. The reflection by metal NPs can be regarded as arising from a portion of the light scattered by the metal NPs. Fig. 3b shows the simulated extinction spectra of a single hemisphere Au NP (60 nm in diameter) on quartz when light is incident from front and back sides. Extinction peaks at approximately 600 nm are present in both spectra regardless of the incident direction of the light. However, the peak intensities are different. When light is incident from the front side, the extinction peak intensity is smaller than that when light is incident from the back side. At the LSP resonance wavelength, the extinction peak intensity when light is incident from the back side is approximately 1.5 times that when light is incident from the front side.

This behavior can be explained by the different local driving field intensities 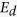 at the position of the Au sphere when light is incident from different directions. When light is incident to the air/NPs/substrate, the local driving field intensities can be regarded as superposition of field intensities of incident wave and reflecting wave as shown in Fig. 3a. Thus we obtain 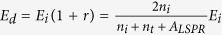  , where 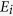 is the electric field intensity of incident light. Then we can obtain 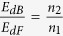, where 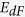 and 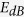 are the local driving field intensities of LSPs when light is incident from front and back side respectively, 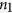 and 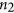 are the refractive indices of the materials above and beneath the Au sphere respectively. Despite the different LSP resonance wavelengths caused by the different effective refractive indices beneath the NPs, one can see from Fig. 3b–d that the ratio of the simulated extinction peak intensities when light is incident from back and front 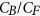 is equal to 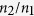.

**Figure 4:**
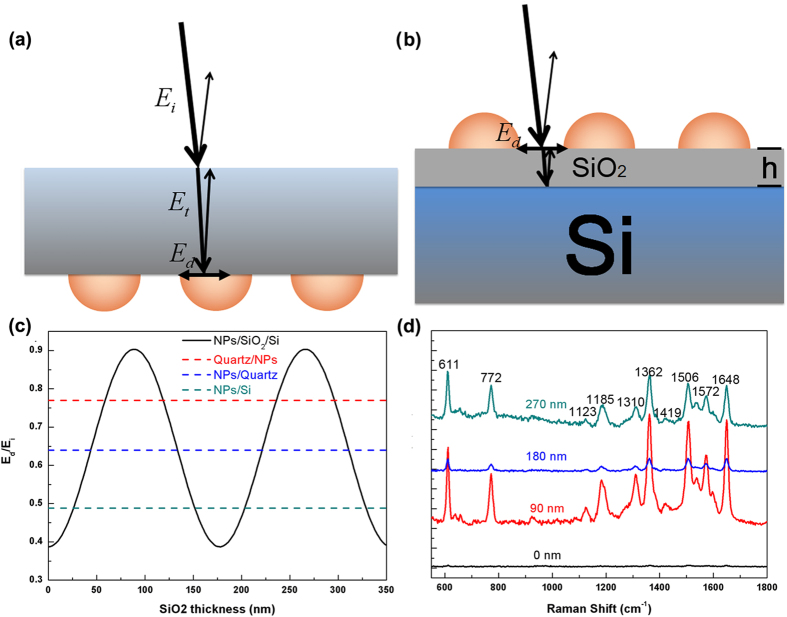
Additional near field enhancement. (**a**) Schematic illustration of local driving field of Au NPs on substrate when light is incident from back side. (**b**) Schematic illustration of local driving field of Au NPs/SiO2/Si when light is incident from front side. (**c**,**d**) illustrate the local driving field intensity and SERS spectra of R6G on Au NPs/SiO2/Si structures as a function of SiO2 thickness.

For near field applications, one can see from Fig. 3a that when light is incident from the back side, the LSP resonance can be additionally enhanced by the enhancement of local driving field by the LSPs. F. J. Beck *et al.* and S. Jayawardhana *et al.* have developed back incident technique based on this behavior^28^,^29^. In this way, the efficiencies of the LSPs enhanced solar cells and the intensity of the surface-enhanced Raman scattering (SERS) signal have been significantly enhanced. However, if light is not generated from the devices, the energy loss by reflection at the interface is unavoidable, for example, photodetectors, solar cells, and most TERS, SERS setups. When light is incident from air and the metal NPs are located at the back side of a thick non-absorbing dielectric film, as shown in Fig. 4a, the local driving field intensity should be 
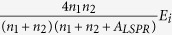
, where 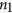 and 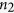 are the refractive indices of air and substrate respectively. Although this local driving field intensity of LSPs is larger than that when light is incident from front side, which equals to 
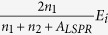
, it is still not an optimized structure. Considering the structure shown in Fig. 4b, when metal NPs are separated from a dielectric medium with high refractive index 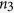 by a thin film with low refractive index 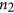, the local driving field intensity of LSPs can be written as 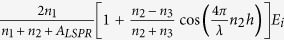, where *h* is the thickness of the thin film, and 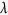 is the wavelength of the incident wave. One can see that when 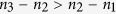, the local driving field intensity of LSPs shown in Fig. 4b will be higher than that shown in Fig. 4a. Figure 4c shows the local driving field intensity of LSPs as a function of *h*, where *A*_*LSPR*_, 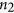 and 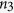 are set as 0.6, 1.5 and 3.5 respectively, 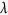 equals to 532 nm. One can see that when 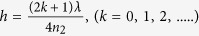, the local driving field intensity of LSPs is maximized. Figure 4d presents SERS results of R6G molecules using Au NPs/SiO_2_/Si as substrates. One can see that when the thickness of SiO_2_ equals to 90 nm and 270 nm, the SERS signals are much larger than when the thickness of SiO_2_ equals to 0 nm and 180 nm.

## Discussion

In summary, we observed the asymmetric light reflectance phenomenon in metallic NPs fabricated on quartz substrate. The difference of the reflectivity when light is incident from different directions can be attributed to the superposition of waves reflected from metallic NPs and from the dielectric medium interface. A modified Fresnel coefficient model indicates that the phase shift of the wave reflected from metal NPs should be π. Thus the superposition between the reflected waves from metallic NPs and dielectric medium interface creates either constructive or destructive interference when light is incident from media with lower or higher refractive indices, respectively. Theoretical analysis and FDTD simulation suggest that this behavior can achieve zero reflectance via adjusting the density of metal NPs that can enhance the sensitivity of LSP sensors. Near field FDTD simulation shows that the ratio of the extinction peak intensities when light is incident from different directions equals the ratio of the refractive indices of two mediums beside the interface, implying that when light is incident from the medium with higher refractive index, metallic nanostructures would have higher coupling efficiency with the incident light. This behavior can be attributed to the different local driving field according to the Fresnel equation. Further investigating shows that the LSPs coupling efficiency when light is incident from air can be regulated by separating the metallic NPs from substrate using a low refractive index thin film. The highest LSPs coupling efficiency is achieved when the thickness of the thin film equals to 
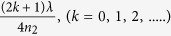
. This work provides a general method to optimize the LSPs coupling efficiency that may improve the performance of LSP based devices such as LEDs, photodectors and solar cells or techniques such as SERS, TERS and chemical sensing.

## Methods

### Sample Fabrication

Au and Ag NPs were fabricated on quartz wafers of 0.5 mm thickness. The quartz wafers were ultrasonically degreased in acetone, ethanol and then double deionized water for 3min each. Au and Ag films with thickness of approximately 2 nm were sputtered by using SCD005 (Balzers Union, Balzers, Liechtenstein). The sample was then annealed in N_2_ ambient by using the RTA device at 450 °C for 60 s to form Au and Ag NPs. The average sizes of fabricated Au and Ag NPs are 28 and 20 nm respectively. The densities of Au and Ag NPs are 8.5 × 10^10^ and 4.5 × 10^10^ cm^–2^ respectively. For SERS measurement, SiO_2_/Si wafers with different SiO_2_ thickness were used as substrates to fabricate Au NPs. The thickness of dry-oxidized SiO_2_ layers was 0 nm, 90 nm, 180 nm and 270 nm respectively. Au film with thickness of approximately 2 nm was then sputtered and followed by annealing in N_2_ ambient by using the RTA device at 700 °C for 60 s to form Au NPs.

### Measurements and Simulations

The optical characterizations of transmittance and reflectance spectra were performed using a UV-Vis-NIR spectrophotometer (Varian Cary 5000). All simulations in this work were performed with commercial Lumerical FDTD solutions (version 7.5) software. The incident plane wave propagated perpendicular to the interface of two media from *z* or –*z* directions with the same incident energy density. The polarization direction of incident wave is along the *x*-direction. The refractive index of quartz was set as 1.5.

SERS spectra were acquired using a confocal Raman system (Xplora, Horiba) using 532 nm laser excitation. The laser power was 5mW for the SERS measurements. The typical exposure time for our measurements was 20 s. All the spectra are presented after baseline correction by a polynomial fitting method. The SERS analysis probe R6G was dissolved in DI water to a concentration of 10^−4^ mol/L. The samples were soaked in the R6G solution for 1h. Then the samples were taken out and rinsed using DI water followed by drying in N_2_ gas.

## Additional Information

**How to cite this article**: Huang, K. *et al.* Asymmetric light reflectance from metal nanoparticle arrays on dielectric surfaces. *Sci. Rep.* **5**, 18331; doi: 10.1038/srep18331 (2015).
